# Rituximab Decreases Lymphoproliferative Tumor Formation in Hepatopancreaticobiliary and Gastrointestinal Cancer Patient-Derived Xenografts

**DOI:** 10.1038/s41598-019-42470-w

**Published:** 2019-04-11

**Authors:** Jennifer L. Leiting, Matthew C. Hernandez, Lin Yang, John R. Bergquist, Tommy Ivanics, Rondell P. Graham, Mark J. Truty

**Affiliations:** 1 Department of Surgery, Mayo Clinic, 200 1st St SW, Rochester, MN 55902 USA; 2 Center for Individualized Medicine, Mayo Clinic, 200 1st St SW, Rochester, MN 55902 USA; 3 Department of Surgery, Henry Ford Medical Center, 2799 West Grand Blvd, Detroit, MI 48202 USA; 4 Department of Pathology, Mayo Clinic, 200 1st St SW, Rochester, MN 55902 USA

## Abstract

High engraftment rates are critical to any patient-derived xenograft (PDX) program and the loss of PDX models due to the development of lymphoproliferative tumors (LTs) is costly and inefficient. We hypothesized that routine injection of rituximab, an anti-CD20 antibody, at the time of implantation would reduce the incidence of LTs. Rituximab injection was added to the standard PDX engraftment protocol. Univariate analysis and multivariate logistic regression were used to determine the significance of various factors. A total of 811 generations of PDX were implanted with 406 receiving rituximab with implantation. On multivariable analysis, rituximab was an independent factor for decreased LT formation across the entire cohort (OR 0.465, 95% CI 0.271–0.797, p = 0.005). Hepatocellular carcinomas (OR 0.319, 95% CI 0.107–0.949, p = 0.040) and cholangiocarcinomas (OR 0.185, 95% CI 0.049–0.696, p = 0.113) were the specific malignant histologic subtypes that demonstrated the greatest benefit. The frequency of LTs decreased across the entire cohort with rituximab administration and PDX tumors that are traditionally associated with higher rates of LT formation, HCCs and CCAs, appear to benefit the most from rituximab treatment. Routine use of rituximab at the time of tumor implantation may have significant programmatic benefits for laboratories that utilize PDX models.

## Introduction

Patient-derived xenografts (PDX) are clinically relevant translational models that accurately recapitulate individual patient tumor histopathologic and molecular phenotypes^1,2^. Their roles in individualized oncologic research are myriad and are most commonly utilized in the preclinical setting for translational applications. They can predict a patient’s response to treatment regimens as well as provide additional tissue that can be used in downstream analyses like whole genome mate-pair sequencing^3–6^. Other traditional and highly utilized preclinical cancer models such as established tumor cell lines and transgenic mice do not provide this level of individuality^7^. Maintaining a high engraftment rate is critical for any PDX program. Inefficiencies stem from primary engraftment failure, when the implanted tumor fails to grow in the murine model, or due to the development of lymphoproliferative tumors (LTs)^8^.

LTs are tumors of lymphocytic origin that are distinctly different from the primary patient tissue both grossly and histologically^9^. The majority of these LTs have been found to be of human origin (CD45+), B-cell phenotype (CD20+ and CD3−), and infected with Epstein-Barr virus (EBV) though others have been of T-cell phenotype or of mouse origin^10–13^. The etiology of these LTs after PDX implantation is not completely understood. Some have suggested that they result from an activation and overgrowth of tumor-infiltrating lymphocytes that are present in the primary patient tissue^14^. Others propose this is due to an activation and overgrowth of latent EBV in the implanted tumor tissue now engrafted in the immunocompromised environment of the murine model^15,16^. The ubiquity of EBV infections, being found in over 90% of the human population, as well as its preference for memory B-cells, the most frequently isolated cell type in LTs, supports this hypothesis^17^. Regardless of etiology, the development of LTs can profoundly contaminate the inventories and subsequent downstream analyses of any high volume PDX program, and methods to decrease their incidence are critically needed^11^.

Rituximab is a monoclonal anti-CD20 antibody that causes B-cell depletion and is currently FDA approved for the treatment of CD20-positive hematopoietic malignancies such as Chronic Lymphocytic Leukemia and Non-Hodgkin Lymphoma^18,19^. The use of this antibody was recently shown to decrease the rates of LTs in an ovarian cancer PDX program^13^. We hypothesized that routine administration of rituximab would similarly decrease the rate of LT formation in our hepatopancreaticobiliary (HPB) and gastrointestinal (GI) cancer PDX models.

## Results

**Figure 1:**
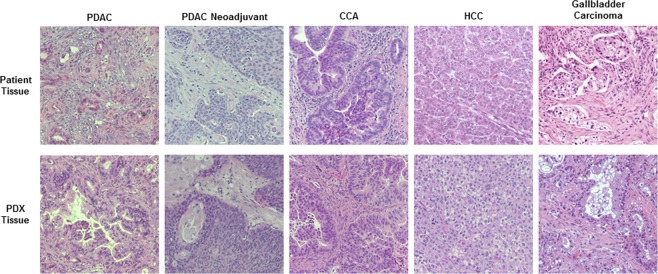
Establishment of PDX Models. Representative samples of the five main tumor subtypes: PDAC, PDAC neoadjuvant, CCA, HCC, and gallbladder carcinoma as one of the miscellaneous GI tumors. Top row is primary patient tissue with the bottom row showing a corresponding PDX tissue that was generated from the primary tissue. (PDAC: pancreatic ductal adenocarcinoma, CCA: cholangiocarcinoma, HCC: hepatocellular carcinoma, GI: gastrointestinal).

**Figure 2:**
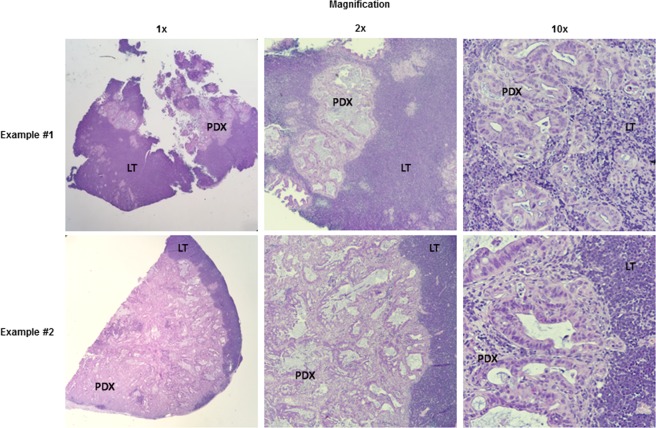
LT formation. Two examples of LT formation are seen above. In Example #1 (top row), small nests of PDX tumor tissue are seen being completely surrounded by lymphocytes. In Example #2 (bottom row), lymphocytes are seen at the edge of the specimen with a clear differentiation between the PDX tumor tissue and the LT. (LT: lymphoproliferative tumor, PDX: PDX tumor tissue).

From 2013–2018, 338 unique patient tumors were implanted in a total of 811 generations. Four-hundred five (49.9%) underwent standard implantation while four-hundred six (50.1%) underwent implantation with rituximab administration. Other than the use of rituximab, there were no other changes to implantation techniques during this time period. There were no complications with the use of rituximab and mice tolerated the injection without difficulty. Histologic verification was performed on all PDX models to ensure recapitulation of the primary patient tissue (Figs 1 and 2). The most common tumor subtype was pancreatic ductal adenocarcinomas (PDAC) after neoadjuvant therapy (n = 208), followed by cholangiocarcinomas (CCA) (n = 193), miscellaneous GI tumors (n = 149), treatment naïve PDACs (n = 142), and hepatocellular carcinomas (HCC) (n = 119). Overall successful engraftment rate was 46.1% (n = 374). Treatment naïve PDACs had the highest success rate with 56% successfully engrafting (80 out of 142), followed by miscellaneous tumor GI tumors (77 of 149, 52%), neoadjuvant PDACs (103 of 208, 50%), CCAs (96 of 193, 50%), and HCCs (18 of 119, 15%).

**Figure 3:**
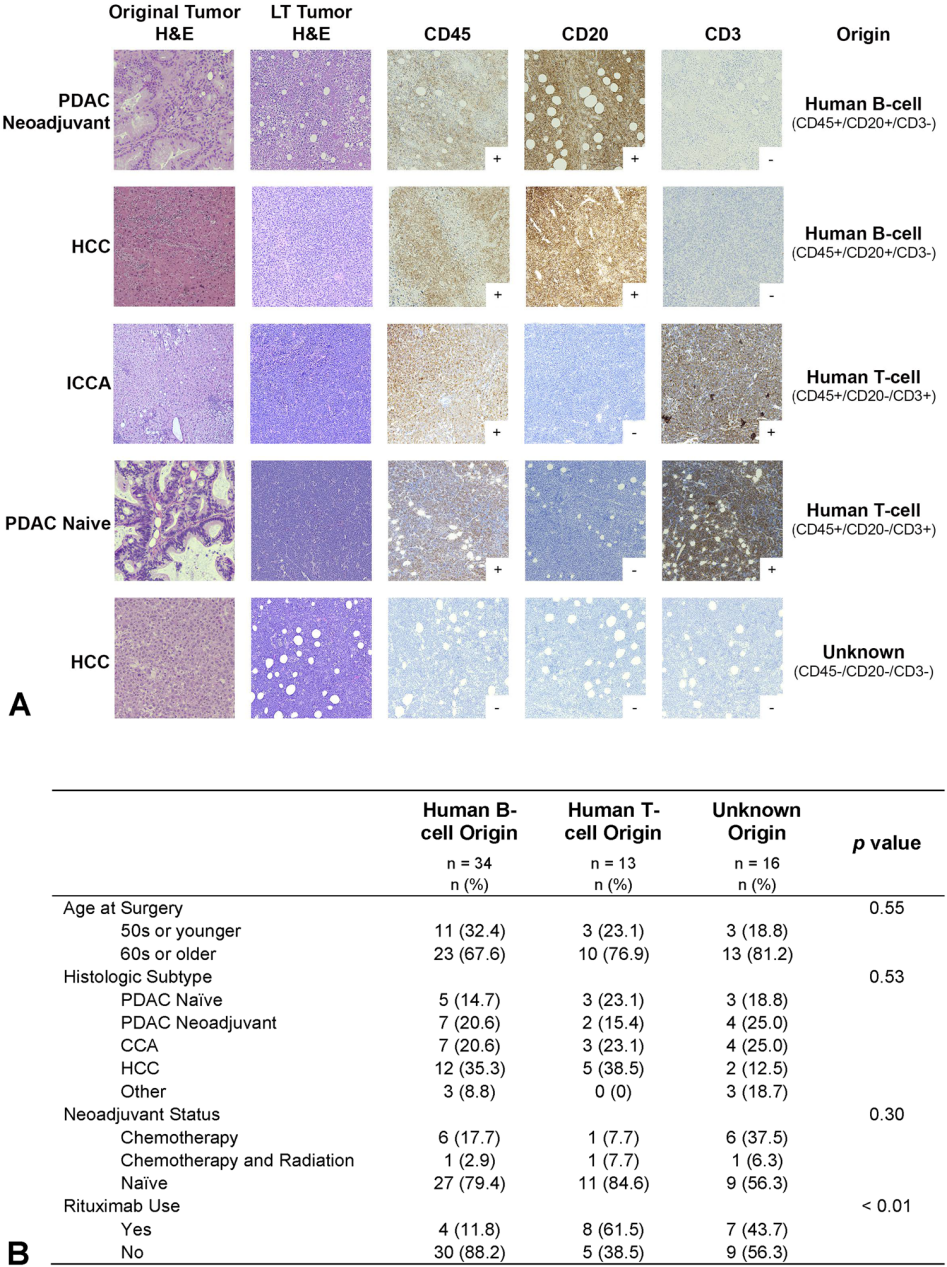
LT characterization. Characterization was accomplished by performing IHC for CD45 (human origin), CD20 (B-cell origin), and CD3 (T-cell origin) with representative tumors shown (**A**). Univariate analysis of difference between human B-cell, human T-cell, and unknown LTs. (H&E: hematoxylin and eosin, PDAC: pancreatic ductal adenocarcinoma, HCC: hepatocellular carcinoma, ICCA: intrahepatic cholangiocarcinoma, HCC: hepatocellular carcinoma).

The overall rate of LTs across the entire cohort was 9.7% (n = 79). Characterization with IHC was able to be performed on 63 (80%). Of these, 34 (54%) were human B-cell origin (CD20+), 13 (21%) were human T-cell origin (CD3+), and 16 (25%) were of unknown origin, with possible mouse origin (CD45−/CD20−/CD3−) (Fig. 3A). Univariate analysis of these tumor types showed the only significant difference to be rituximab administration, with only 4 (12%) human B-cell LTs having received rituximab at implantation (Fig. 3B).

Patient, tumor, and xenograft characteristics are listed in Table 1. Univariate analysis found a number of characteristics to be significant as it relates to LT formation. These included patient age at surgery (p = 0.001), tumor subtype (p = 0.008), patient neoadjuvant status (p < 0.001), tumor differentiation (p = 0.037), perineural invasion (PNI) (p = 0.009), lymphovascular invasion (LVI) (p = 0.018), administration of rituximab (p = 0.003), and PDX generation (p = 0.022). However, on multivariable analysis, the administration of rituximab (OR 0.465, 95% CI 0.271–0.797, p = 0.005) and having received both chemotherapy and radiation in the neoadjuvant setting (OR 0.093, 95% CI 0.024–0.353, p = 0.005) were independent factors associated with a decreased rate of LT formation while being 70 years of age or older at the time of surgery (OR 3.463, 95% CI 1.203–9.965, p = 0.021) and being in a subsequent mouse generation (OR 2.037, 95% CI 1.175–3.531, p = 0.011) were independent factors that were associated with an increased rate of LT formation (Table 2).

**Table 1:** Overall patient, tumor, and PDX characteristics.

	No LT		LT		p
	n = 732		n = 79		
	n	%	N	%	
Patient Sex					0.055
Male	435	59.43	38	48.1	
Female	297	40.57	41	51.9	
Age at Surgery					**0.001**
40s or younger	121	16.53	5	6.33	
50s	153	20.9	20	25.32	
60s	261	35.66	19	24.05	
70s or older	197	26.91	35	44.3	
Tumor Subtype					**0.008**
PDAC Naïve	126	17.21	16	20.25	
PDAC Neoadjuvant	192	26.23	16	20.25	
CCA	176	24.04	17	21.52	
HCC	97	13.25	22	27.85	
Other	141	19.26	8	10.13	
Neoadjuvant Status					**<0.001**
Chemotherapy	123	15.17	17	21.52	
Radiation	3	0.41	0	0	
Both	161	21.99	3	3.8	
Naïve	445	60.79	59	11.71	
Differentiation					**0.037**
Well	84	11.49	6	7.59	
Moderate	350	47.88	47	59.49	
Poor	255	34.88	26	32.91	
Unknown	42	5.75	0	0	
Lymph Node Status					0.792
Negative	309	42.21	35	44.3	
Positive	269	36.75	26	32.91	
Unknown or N/A	154	21.04	18	22.78	
Margin Status					0.095
Negative	594	81.15	69	87.31	
Positive	56	7.65	7	8.86	
Unknown or N/A	82	11.2	3	3.8	
PNI					**0.009**
Negative	346	47.27	50	63.29	
Positive	283	38.66	25	31.65	
Unknown or N/A	103	96.26	4	5.06	
LVI					**0.018**
Negative	439	59.97	55	69.62	
Positive	190	25.96	21	26.58	
Unknown or N/A	103	14.07	3	3.8	
Rituximab					**0.003**
No	353	48.22	52	65.82	
Yes	379	51.78	27	34.18	
Mouse Generation					**0.022**
First	315	43.03	23	29.11	
Subsequent	417	56.97	56	70.89	
Mouse Strain					0.523
NOD/SCID	672	91.8	71	89.87	
NSG	60	8.2	8	10.13	

LT: lymphoproliferative tumor, PDAC: pancreatic ductal adenocarcinoma, CCA: cholangiocarcinoma, HCC: hepatocellular carcinoma, PNI: perineural invasion, LVI: lymphovascular invasion.

**Table 2:**
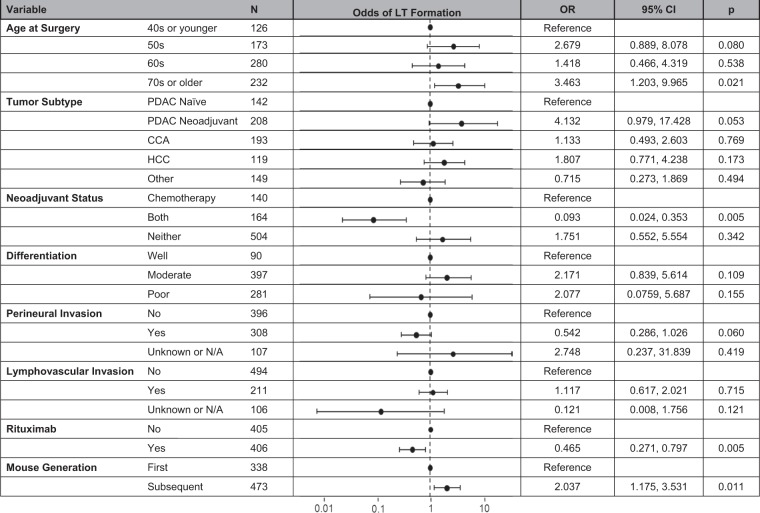
Adjusted odds of LT formation for entire cohort.

In the F1 cohort, the only significant factor related to the frequency of LT formation was the administration of rituximab (OR 0.231, 95% CI 0.067–0.794, p = 0.013) (Table 3). On univariate analysis, significant factors associated with LT formation in subsequent generations of mice included mouse generation (p = 0.047), tumor subtype (p = 0.047), and rituximab use (p = 0.013). However, rituximab administration was the only independent factor on multivariable analysis and it was associated with decreased LT rates (OR 0.445, 95% CI 0.245–0.807, p = 0.008) (Table 4).

**Table 3:** Initial implantation cohort characteristics.

	No LT		LT		p	OR (95% CI)
	n = 315		n = 23			
	n	(%)	n	(%)		
Patient Sex					1.000	—
Male	177	56.19	13	56.52		
Female	138	43.81	10	43.48		
Age at Surgery					0.941	—
40s or younger	43	13.65	2	8.7		
50s	65	20.63	5	21.74		
60s	115	36.51	8	34.78		
70s or older	92	29.21	8	34.75		
Neoadjuvant Status					0.157	—
Chemotherapy	47	14.92	6	26.09		
Radiation	1	0.32	0	0		
Both	81	25.71	2	8.7		
Neither	186	59.05	15	65.22		
Tumor Subtype					0.177	—
PDAC Naïve	52	16.51	2	8.7		
PDAC Neoadjuvant	94	29.84	6	26.09		
CCA	76	24.13	6	26.09		
HCC	38	12.06	7	30.43		
Other	55	17.46	2	8.7		
Differentiation					0.852	—
Well	37	11.75	3	13.04		
Moderate	161	47.63	13	56.52		
Poor	98	31.11	7	30.43		
Unknown	19	6.003	0	0		
Lymph Node Status					0.120	—
Negative Nodes	133	42.22	6	26.09		
Positive Nodes	116	36.83	8	34.78		
Unknown	66	20.95	9	39.13		
Margin Status					0.915	—
Negative Margin	257	81.59	19	6.88		
Positive Margin	23	7.3	2	8.7		
Unknown or N/A	35	11.11	2	8.7		
PNI					0.130	—
No	148	46.98	16	69.57		
Yes	122	38.73	5	21.74		
Unknown or N/A	45	14.29	2	8.7		
LVI					0.633	—
No	197	62.54	14	60.87		
Yes	71	22.54	7	30.43		
Unknown or N/A	47	14.92	2	8.7		
Ischemic Time					0.650	—
<60 minutes	173	54.92	11	47.83		
>/=60 minutes	132	41.9	11	47.83		
Unknown	10	3.17	1	4.35		
Rituximab					**0.013**	**0.231**
No	191	60.63	20	86.96		(0.067- 0.794)
Yes	124	39.37	3	13.04		
Mouse Strain					0.468	—
NOD/SCID	286	90.79	20	86.96		
NSG	29	9.21	3	13.04

**Table 4:**
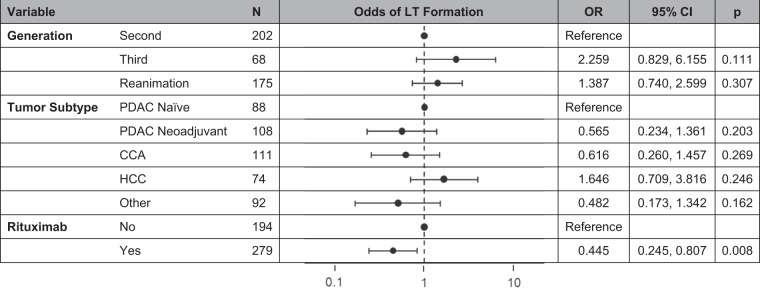
Adjusted model for odds of LT formation in subsequent generations.

On subgroup analysis of tumor subtypes, rituximab was found to be the only factor independently associated with LT formation in HCCs (OR 0.319, 95% CI 0.107–0.949, p = 0.040) and CCAs (OR 0.185, 95% CI 0.049–0.696, p = 0.013). Rituximab administration was not significant after multivariable analysis for neoadjuvant PDACs and was also not significant for naïve PDACs or miscellaneous GI tumors (Table 5). A summary of the effect of rituximab on the odds of LT formation is seen in Table 6.

**Table 5:** Tumor subtypes and the impact of rituximab administration.

	Univariate Analysis					Multivariable Analysis		
	No LT		LT		p	OR	95% CI	p
	n = 732		n = 79					
	n	%	n	%				
PDAC Naïve					0.529	—	—	—
No rituximab	82	65.08	6	37.50				
Rituximab	44	34.92	10	62.50				
PDAC Neoadjuvant					**0.017**	0.305	0.075, 1.239	0.097
No rituximab	93	48.44	13	81.25				
Rituximab	99	51.56	3	18.75				
CCA					**0.020**	0.185	0.049, 0.696	**0.013**
No rituximab	91	51.70	14	82.35				
Rituximab	85	48.30	3	17.65				
HCC					**0.034**	0.319	0.107, 0.949	**0.040**
No rituximab	37	38.14	14	63.64				
Rituximab	60	61.86	8	36.36				
Misc GI Tumors					0.146	—	—	—
No rituximab	50	35.46	5	62.50				
Rituximab	91	64.54	3	37.50

**Table 6:**
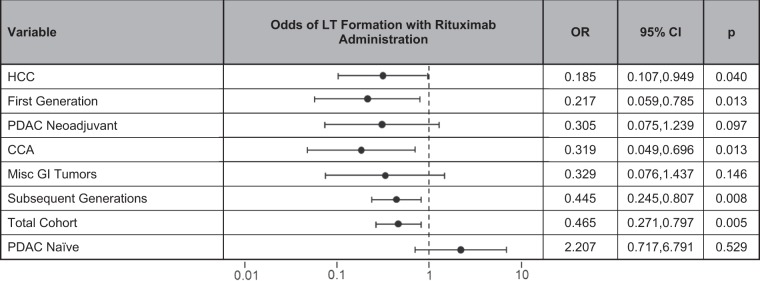
Influence of rituximab on LT formation.

## Discussion

The purpose of this study was to determine whether the addition of rituximab to the engraftment protocol would decrease the rates of LTs in our HPB and GI cancer PDX models in order to avoid detrimental contamination of our inventory. We found that a single dose of rituximab at the time of engraftment was independently associated with decreased LT formation across the entire cohort. That benefit was seen in further subgroup analysis for initial implantations, subsequent implantations, and was most effective for CCA and HCC tumor histology.

In addition to the administration of rituximab, a number of other factors were found to be significant in the formation of LTs. The odds of LT formation were significantly increased in patients seventy years of age or older at the time of implantation in our overall cohort. There are significant changes and remodeling of the immune system as a person ages that likely influence both the formation of cancers^20,21^ and the resulting tumor biology^22,23^. Some of these factors include decreased antibody production, increased memory B cells, and decreased T cell function^24^ which may play a role in the increased formation of LTs in this population. Patients who received both chemotherapy and radiation in the neoadjuvant setting were significantly less likely to develop LTs in the PDX model. Neoadjuvant chemotherapy and radiation therapy have been shown to increase immune cell populations in a number of different malignancies including rectal, gastric, and breast cancer^25–28^. They also appear to be able to change the subsets of lymphocytes that are present after therapy^29^ which may be a factor in LT formation. Lastly, being from a subsequent generation, rather than the first generation, increased the odds of LT formation which is likely due to the fact that histological verification takes time and implantation into subsequent generations (F2 and beyond) has likely already occurred when an LT is confirmed on histology.

Rituximab administration had varying effects on the rate of LT formation depending on tumor subtypes. It represented an independent factor for decreased LT formation in HCCs and CCAs but did not significantly influence their formation in PDAC, both naïve and neoadjuvant, or the group of miscellaneous GI tumors. The cause for this is unclear though it may relate to the density and composition of lymphocytes in the primary tissue of origin. HCCs had the highest percentage of LTs out of the five tumor subtypes and other groups have had difficulty establishing HCC PDX models given the high rate of LT formation^16^. The liver is an important organ in the immune system^30^ and has one of the highest densities of immune cells^31^ which may contribute to the high rate of LT formation. Along those lines, a group of investigators found that increased lymphocytic infiltration from chronic inflammation in the setting of gastric cancer increased their rates of gastric cancer PDX LT formation^32^, though another study did not find a correlation between leukocyte infiltration of the parent HCC tissue and the development of LTs^16^.

There is a growing interest in methods to decrease the rates of LT formation within a PDX program. One method requires early identification based on growth pattern and gross appearance. In our experience, LTs tend to grow very rapidly, are very soft in texture, and are pale and fleshy in appearance. Avoiding implantation of these suspicious tumors until their histologic verification could prevent unnecessary waste but it would not decrease the rate of LTs in primary generations. Another recommendation has been to quantify EBV RNA in samples and considering not implanting those with high levels due to an increased likelihood of progression to a LT^11^. The disadvantage of such an approach is a delay in implantation, which currently takes place immediately following surgical resection. This increases cold ischemia time and may negatively impact engraftment rates. Rituximab administration decreases the rates of LTs in primary generations while also allowing for immediate implantation.

The type of murine host has been implicated in contributing to the rate of LT formation. Nude mice, who lack only T-cell function, were one of the first immunodeficient mouse strains to be used for cancer models but the engraftment rate of implanted tumors was low^33^. NOD/SCID mice lack a B-cell response with limited natural killer (NK) cell function while NOD scid gamma (NSG) and NOD/Shi-scid/IL-2Rϒ^null^ (NOG) are further immunosuppressed and lack NK cell function entirely^2,11,34^. It has been shown that nude mice either do not form LTs or do not form LTs at the same rate when compared to the more severely immunodeficient strains^13,32^. More severely immunodeficient strains have demonstrated better engraftment rates when compared to nude mice^2,12^. Therefore, it is believed that improved engraftment rates may come at the price of increased LT rates and other infections^9^. However, we did not see any significant differences in LT formation between NOD/SCID and NSG mice in our cohort.

This study is limited by a lack of randomization and no control group. Implantations were done by a variety of laboratory personnel over a two year period, and despite standardization of implantation methodology, this does not account for individual differences that may play a role in PDX outcomes.

Routine rituximab administration is a safe and efficient way to decrease the rate of LT formation in a HPB and GI cancer PDX program. The benefit of reducing LT formation is preventing detrimental and costly contamination to a large inventory while preserving the integrity of subsequent downstream analyses. We have incorporated this in our standard engraftment protocol as a way to increase efficiency and limit waste in our program.

## Methods

With Mayo Clinic Institutional Review Board (IRB) and Institutional Animal Care and Use Committee (IACUC) approval and in accordance with established guidelines and regulations, PDX models were generated according to a previously established protocol^35^. In brief, informed consent was obtained from patients prior to participation in the study. Surplus tumor tissue and adjacent normal tissue was collected immediately following surgical resection from the frozen section pathology lab once a diagnosis of cancer had been verified by a pathologist. This tissue was immediately placed into serum free Roswell Park Memorial Institute 1640 (Invitrogen, Carlsbad, CA) media that was stored on ice. Under sterile conditions, the tumor tissue was sectioned with a scalpel into 2–3 mm^3^ sections and submerged in 300 uL of MatriGel (Corning, Corning, NY) on a sterile petri dish. Six to eight week old male and female nonobese diabetic severe combined immunodeficiency (NOD/SCID) mice (Department of Comparison Medicine, Mayo Clinic, Rochester) were anesthetized with Isoflurane. The flanks of the mice were sterilized with 70% ethanol and two small incisions were made on the bilateral flanks. Small bilateral subcutaneous pockets were made bluntly and a piece of tumor was placed into the subcutaneous pocket. VetBond (3 M, Maplewood, MN) was used to close the wounds and the mice were monitored for complications following the procedure. The remaining original patient tumor tissue was cryopreserved and is referred to as the F0 tissue. Mice are monitored for tumor growth and signs of conditional decline. Specifically, mice that develop LTs in our experience generally have a rapid tumor growth with worsening clinical features including, hair loss, hunching, and weight loss.

Beginning in January 2016, rituximab administration was added to the engraftment protocol and used for the implantations that followed. An intraperitoneal injection of 1 mg (100 μL) of rituximab was administered immediately preceding tumor implantation. Once growing tumors were confirmed and reached a size of approximately 1000 mm^3^, or when an IACUC established endpoint was met, the tumors were harvested. These endpoints included tumors greater than or equal to 10% body weight, ulcerated tumors, inability of the mouse to ambulate, or weight loss greater than or equal to 20% of their body weight. Each PDX tumor was histologically verified by a GI pathologist by comparing it to the original patient tumor from which it was derived. When there is a tumor that is suspicious for an LT, in addition to hematoxylin and eosin staining, immunohistochemistry for anti-cytokeratin OSCAR is done as this should be absent in LTs. The tissue obtained from the first generation of mice is referred to as the F1 generation, or the initial implantation cohort.

When the first F1 tumor from a generation of mice was harvested, it was subsequently implanted into 5 new mice using the same procedure described above. This is done to confirm that the PDX model is able to grow and be passed *in vivo*. These mice were again monitored for tumor formation and harvested when any of the previously listed criteria were met. The second generation of mice is referred to as the F2 generation. If additional tissue was needed, F2 tumors were implanted into additional subsequent generations, and were classified according to which generation of mice they were a part of (F3 = third generation, F4 = fourth generation, and so on). The term subsequent generations refers to any F2 generation or higher.

When a specific patient tumor was needed but there were no mice currently with the tumor in the inventory, the model was reanimated from cryopreserved tissue. This reanimation process involved thawing previously cryopreserved tissue, washing the tissue with sterile room-temperature phosphate-buffered saline (PBS), and implanting according to the above protocol.

Characterization of the LTs was done using standard immunohistochemistry (IHC) and was performed at the Pathology Research Core (Mayo Clinic, Rochester, MN) using the Leica Bond RX stainer (Leica). The following antibodies were used: CD45 for human origin (Clone 2B11&PD7/26; Dako), CD3 for T-cells (Clone F7.2.38; Dako), and CD20 for B-cells (Clone L26; Dako). Specimens were scored in a blinded fashion.

A retrospective analysis was done for all HPB and GI cancers that underwent PDX engraftment. Patient, tumor, and PDX characteristics were compared between LT generations and generations with no LT formation. Chi squared and Fisher exact test were used for univariate statistical comparison of categorical variables. Multivariable logistic regression was used to determine the odds ratio (OR) for statistically significant univariate factors. An alpha level of 0.05 was considered significant. JMP software was used to analyze all the data (JMP^®^ Pro, Version 13.0.0, SAS Institute Inc., Cary, NC, USA).

## Data Availability

The dataset generated and analyzed during the current study is not publicly available because of privacy concerns but is available from the corresponding author on reasonable request.
